# First report of Bickerstaff’s brainstem encephalitis caused by *Salmonella Dublin*: a case report

**DOI:** 10.1186/s12883-021-02230-8

**Published:** 2021-05-15

**Authors:** Jiangbo Xie, Tingting Zhang, Tao Liu

**Affiliations:** Department of Neurology, Weifang Traditional Chinese Hospital, Weifang, China

**Keywords:** Bickerstaff’s brainstem encephalitis, *Salmonella Dublin*, Case report

## Abstract

**Background:**

Diseases caused by nontyphoid Salmonella can range from mild, to self-limiting gastroenteritis and severe invasive infection. Relatively rarely, Salmonella may cause severe encephalopathy.

**Case presentation:**

We report a suspected case of Bickerstaff’s brainstem encephalitis caused by *Salmonella Dublin*. A young man presented with impaired consciousness, ataxia, dysarthria, limb weakness, and restricted eyeball abduction. His clinical symptoms were consistent with Bickerstaff’s brainstem encephalitis.

**Conclusions:**

This is the first case report of Bickerstaff’s brainstem encephalitis caused by *Salmonella Dublin* in the literature. After treatment, he recovered and was discharged. Early antibiotic treatment of sepsis may control the disease and avoid serious encephalopathy.

## Background

Most Salmonella species that are pathogenic in humans belong to *Salmonella Enteritidis*. *Salmonella Typhi* and *Salmonella Typhi* A are the pathogens that cause typhoid fever, a potentially fatal disease. Diseases caused by nontyphoid Salmonella can range from mild to self-limiting gastroenteritis and severely invasive infections. Salmonella may cause severe encephalopathy, though it is relatively rare. We report a case of Bickerstaff’s brainstem encephalitis (BBE) caused by *Salmonella Dublin*. Cerebrospinal fluid (CSF) examination on admission was normal, as was a brain magnetic resonance imaging (MRI) scan. An anti-GQ1b antibody test was negative, and CSF protein/cell dissociation occurred 20 days after admission. During hospitalization, the patient was treated with intravenous immunoglobulin (IVIg) therapy and then discharged.

## Case presentation

A 33-year-old man developed diarrhoea 1 day after eating fried pork with chili and fever the next day, with a body temperature of 40 °C. The results of blood and faecal cultures were positive for *Salmonella Dublin*. Influenza A virus, influenza B virus, *Mycoplasma pneumoniae*, *Chlamydia pneumoniae*, respiratory syncytial virus, *Haemophilus influenzae*, varicella-zoster virus, Legionella, Campylobacter and SARS-CoV-2 tests were all negative. The patient developed drowsiness after 3 days, with gradual weakening of the limbs, dysarthria, binocular abduction paralysis, and ataxia. His brain MRI scan was normal. After 5 days, the patient could not raise his limbs. These symptoms were accompanied by liver function damage and myocardial damage. After 7 days, the patient still had weakness of the limbs and dysarthria. However, as other indicators improved, he was transferred from the Intensive Care Unit ward to the Neurology ward. Physical examination of the nervous system mainly showed flaccid paralysis of the limbs, disappearance of the bilateral tendon reflex, inability to speak, ataxia (bilateral limb paralysis limited the ability to assess gait), and positive bilateral Babinski signs. At that time, lumbar puncture results for intracranial pressure, CSF protein, and CSF cell number were normal, and the CSF was cultured for 3 days with no bacterial growth. On re-examination, the brain MRI and cervical MRI were normal. As BBE was suspected clinically, IVIg therapy (0.4 g/kg) was given for 5 days, but the patient’s symptoms did not improve significantly. Twenty days after admission, lumbar puncture examination showed that the protein content had increased by 0.86 g/L (the normal range is 0.08–0.43 g/L); the cell number was normal (the number of nucleated cells was 6, normal range 0–8; the red blood cell count was 0), CSF and serum anti-GQ1b antibody was negative, and CSF anti-MOG, anti-AQP4, and anti-MBP antibodies were all negative. The results of electrophysiological examination were normal (14 days and 60 days after onset). Considering no obvious improvement, at 21 days, he was given IVIg again at the same dose for 5 days. The patient’s symptoms gradually improved, and he was able to start walking on his own. His limb collateral movement was significantly better than before, his eye movement was normal, and his voice was low. The patient’s speech could be heard clearly, and he was given speech rehabilitation training. After 3 months, the patient could walk 10 m without assistance but was unable to run (the grade of the GBS disability scale was 2) [1]. His speech also returned to normal.

## Discussion and conclusion

Salmonella causes a tremendous global burden of disease [2]. It is estimated that 535,000 cases of invasive nontyphoid Salmonella infection occur globally each year [3]. The clinical manifestations caused by human salmonellosis are complicated; they can be divided according to severity into gastroenteritis, typhoid fever, sepsis, local purulent infection, and asymptomatic infection. Nontyphoid Salmonella mainly causes self-limiting diarrhoea in healthy individuals, with mild symptoms; blood-borne or focal infections are rare and mostly occur in individuals with specific risk factors [4]. *Salmonella Dublin* is the main pathogen causing Salmonella disease in sheep, cattle, and other animals, though it rarely causes severe symptoms in humans [5]. *Salmonella Dublin* belongs to Group D. Clinically, only 1% of isolates are isolated from faeces; approximately 40% are isolated from blood. The mortality rate is 20% [6]. This case is the first report of BBE caused by *Salmonella Dublin*. There is a previous report of BBE caused by typhoid fever and *Salmonella Paratyphi* A [7, 8].

BBE comprises a group of autoimmune diseases characterized by acute ophthalmoplegia, ataxia, disturbance of consciousness and/or pyramidal tract signs, with an annual incidence rate of less than 0.1 per 100,000 [9]. Although the pathogenesis is still not fully understood, most patients have a history of pre-infection with Guillain–Barré syndrome and Miller-Fisher syndrome, with symptoms such as upper respiratory tract infection and diarrhoea. It has been reported that the main pathogenic microorganisms of BBE are *Mycoplasma pneumoniae* [10–12], *Campylobacter jejuni* [13, 14], cytomegalovirus [15], varicella-zoster virus [7], and Epstein-Barr virus [8] (Table 1). As no evidence of Campylobacter infection was found in our patient’s blood or faeces during hospitalization, we excluded BBE caused by Campylobacter infection. The results of blood culture and faecal culture in our case indicated *Salmonella Dublin* infection. The patient was admitted to the hospital to consider sepsis caused by *Salmonella Dublin*, after which symptoms of encephalopathy appeared. The patient’s typical clinical manifestations and CSF protein/cell dissociation supported the diagnosis of BBE.

**Table 1:** Summary of the characteristics of Bickerstaff’s brainstem encephalitis-related cases reported in the literature

Publisher	Publication time	Country	Infection types	Gender	Age	GQ1bIgG antibody	CSF^a^ test	Imaging examination
Kikuchi,M et al. [10]	1997	Japan	Mycoplasma pneumoniae	male	7	positive	Protein cell dissociation	T2 high signal around aqueduct
Steer AC.et al. [11]	2006	Japan	Mycoplasma pneumoniae	male	11	positive	Protein and cell normal	MRI showed diffuse meningeal enhancement and patchy hyperintensity in the spinal cord
Hussain AM [13]	2007	UK	Campylobacter jejuni	male	54	negative	Protein and cell normal	T2 hyperintense area in the brainstem
Masahiro Mori et al. [14]	2008	Japan	Campylobacter jejuni	female	26	positive	Protein cell dissociation	Not done
Kanzaki A et al. [15]	1995	Japan	Cytomegalovirus	female	17	positive	Protein and cell normal	Brain CT and MRI were normal
Tagawa Y,et a [7]	2000	Japan	Varicella zoster virus	male	59	positive	Protein cell dissociation	No abnormality was found
Rho, YI [8]	2014	Korea	Epstein Barr virus	male	2	negative	Protein and cell normal	MRI was normal
Wali GM.et al. [16]	1991	India	Salmonella typhi	male	14	Not recorded	Protein and cell normal	Brain CT was normal
Bun Sheng et al. [17]	2010	Hong Kong, China	Salmonella Paratyphi A	female	28	positive	Protein and cell normal	brainstem edema with patchy T2 hyperintensity
Gianni Coriolani et al. [12]	2020	Italy	Mycoplasma pneumoniae	Not recorded	7.5	Not recorded	Protein and cell normal	8 mm × 4 mm in T2-FLAIR sequences in the left thalamic area and the posterior left medulla oblongata-spinal

^a^*CSF* Cerebrospinal fluid

Studies have found that anti-GQlb IgG antibodies with the same specificity are prevalent in the serum of BBE patients, and approximately 66% of patients test positive for anti-GQlb IgG antibodies [18]. The titre typically reaches its peak at the beginning of the disease and decreases over time. Notably, there may be other unknown mechanisms or autoimmune targets responsible [18]. Nevertheless, the anti-GQlb IgG antibody test was negative in our case, which may be related to the late examination of the patient.

In most cases of BBE, CSF protein/cell dissociation occurs within 4 weeks of disease development. However, in a small number of cases, the disease is not accompanied by elevated protein levels [12, 13, 15–17]. Brain MRI examination reveals abnormal findings in approximately one-third of BBE patients, with long T2 signal lesions in the brainstem, thalamus, cerebellum, and white matter [18]. Studies have shown that abnormal MRI results are due to vasogenic cerebral oedema [19]. However, the MRI examination in the present case showed no abnormalities.

In our case, the results of blood and stool cultures indicated *Salmonella Dublin* infection, and the patient was admitted to the hospital to consider sepsis caused by this organism. Encephalopathy symptoms appeared days later. The current research mechanism include destruction of the blood–brain barrier via upregulated expression of autocrine MMP-9 in human brain microvascular endothelial cells [20].

Effective treatment for BBE involves immunotherapy, such as steroids, plasma exchange, and IVIg [18, 21]. However, dexamethasone is the first choice for neurological complications caused by Salmonella and Salmonella encephalopathy. Therefore, the clinical recognition of BBE and Salmonella encephalopathy is particularly important, which determines the different treatment options. In this case, the patient’s symptoms gradually improved after IVIg therapy.

In conclusion, we report a case of BBE caused by *Salmonella Dublin*, which needs to be brought to the attention of clinicians. When a patient is suspected of BBE caused by *Salmonella Dublin,*proper immunotherapy is particularly important. Of course, early antibiotic treatment of sepsis may control the disease and avoid severe encephalopathy .

## Data Availability

All data analysed during this study are included in this manuscript.

## Abbreviations

BBE: Bickerstaff’s brainstem encephalitis
IVIg: Intravenous immunoglobulin
MRI: Magnetic resonance imaging
CSF: Cerebrospinal fluid
